# Plea for Detention Hospitals

**Published:** 1901-08

**Authors:** 


A Monthly Review of Medicine and Surgery.

*EDITOR:*

WILLIAM WARREN POTTER, M. D.

All communications, whether of a literary or business nature, books for review and exchanges should be addressed to the editor: 284 FRANKLIN STREET, BUFFALO, N. Y.

VOL. XLI.—LVII. AUGUST, 1901. No. 1.

### Plea for Detention Hospitals.

NO stronger plea for detention hospitals for the suspected insane was ever made than the report in the daily papers of July 5, of the suicide of one person and the attempted suicide of another, confined in the cells of Police Station No. 1. Both patients were insane, so accepted and so confined, and no charge of criminality or of malfeasance was made against them. Both were locked up in prison cells—to await examination and the proper filling out of the commitment papers—before they could be accepted at the Buffalo State Hospital. On the following day the associated press reported a similar occurrence, in New York city—the suicide of an insane man, committed to a prison cell to await examination, no charge of criminality being lodged against him. The lesson of these three cases is that some place is necessary for the temporary lodgement of the violent insane pending examination. It is unsafe to place them behind prison bars, to say nothing of the injustice of such a proceeding. It is unsafe to place these unfortunates under the care of the average police official, even for a single night. It is equally unsafe to leave them at their homes after homicidal or suicidal tendencies have once appeared. The state hospitals are powerless to aid such cases until all legal requirements are fulfilled. No place open, the prison is the only alternative, with results often as the daily papers have chronicled.

In Erie County if detention hospitals are not forthcoming, such cases might be taken to the Erie County Hospital and there detained until the necessary examination is made. Better still would be a small hospital situated in the new county building on the Terrace, fitted up with hospital appliances and managed by physicians, such as Superintendent Long of the Poor Office has repeatedly urged.

Agitation of this question should be kept up until the Board of Supervisors sees and feels the necessity of such a place; in the meanwhile the occurrence in Police Station No. 1, speaks more forcibly than words.

